# Towards reliable myocardial blood-oxygen-level-dependent (BOLD) CMR using late effects of regadenoson with simultaneous ^13^n-ammonia pet validation in a whole-body hybrid PET/MR system

**DOI:** 10.1186/1532-429X-18-S1-O19

**Published:** 2016-01-27

**Authors:** Hsin-Jung Yang, Damini Dey, Jane M Sykes, John Butler, Behzad Sharif, Debiao Li, Sotirios Tsaftaris, Piotr Slomka, Frank S Prato, Rohan Dharmakumar

**Affiliations:** 1 Cedars Sinai Medical Center, Los Angeles, CA, USA; 2 grid.415847.b0000000105562414Lawson Health Research Institute, London, ON Canada; 3 grid.462365.00000000417909464Institute for Advanced Studies Lucca, Lucca, Italy

## Background

BOLD CMR is a non-contrast approach for examining myocardial perfusion but despite major technical advancements to date, its reliability remains weak. A key reason for this is the unpredictable cardiac motion during stress, which can lead to pronounced artifacts that confound/mask the true BOLD signal changes during hyperemia. Recently, regadenoson has become the vasodilator of choice owing to greater patient tolerability and ease of use. We hypothesized that at 10-mins post regadenoson administration (p.r.a), (a) BOLD CMR artifacts at stress are markedly reduced compared to those conventionally acquired at 2-mins p.r.a; and (b) that myocardial perfusion reserve (MPR) remains greater than 2.0 and is highly correlated with the BOLD effects estimated from T_2_ maps.

## Methods

Canines (n = 7) were studied in a PET/MR system. MR acquisitions were used to generate short-axis 2D T_2_ maps; and the PET acquisitions following ^13^N-ammonia infusion were used to quantify myocardial blood flow (MBF). Initially, 2D T_2_ maps and PET signals were acquired at rest. Subsequently, regadenoson (2.5 μg/kg) was administrated. T_2_ maps were acquired at 2- and 10-mins p.r.a and PET signals were acquired at 10-mins p.r.a. Standard deviation (s) of myocardial T_2_ values was measured at rest, 2- and 10-mins p.r.a from T_2_ maps and were used to determine *Myocardial BOLD Variability* (MBV, defined as sT_2_(stress)/sT_2_(rest)) at 2- and 10-min p.r.a. Similarly, using the mean T_2_ values, *Myocardial BOLD Response* (MBR, defined as T_2_(stress)/T_2_(rest)) was computed at 10-mins p.r.a. PET images were analyzed with qPET software to determine MBF and MPR at rest and 10-mins p.r.a and were regressed against MBR

## Results

**Figure 1:**
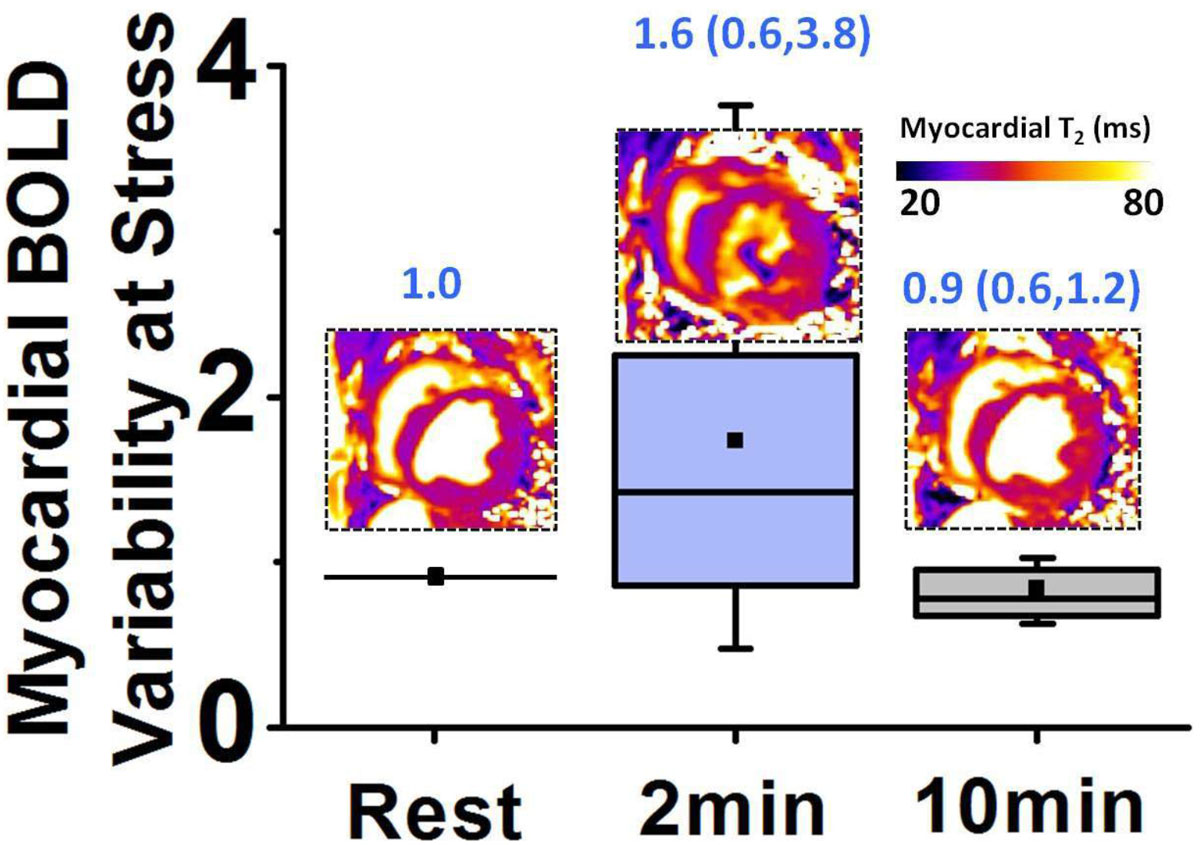
***Myocardial BOLD Variability at Stress Relative to Rest***. Box-plot of Myocardial BOLD Variability (σT_2_ (stress) / σT_2_ (rest)) computed from T_2_ maps acquired at 2 min and 10 min post regadenoson administration (p.r.a) is shown. The mean and range of Myocardial BOLD Variability are shown under various conditions. Large Myocardial BOLD Variability is observed at 2 mins p.r.a. compared to rest and is markedly reduced at 10 mins p.r.a. Representative images acquired at the various conditions are shown for reference. Note the marked T_2_ inhomogeneity in T_2_ image at 2 min p.r.a compared to rest and 10 min p.r.a. σT_2_ denotes the standard deviation of myocardial T_2_.

**Figure 2:**
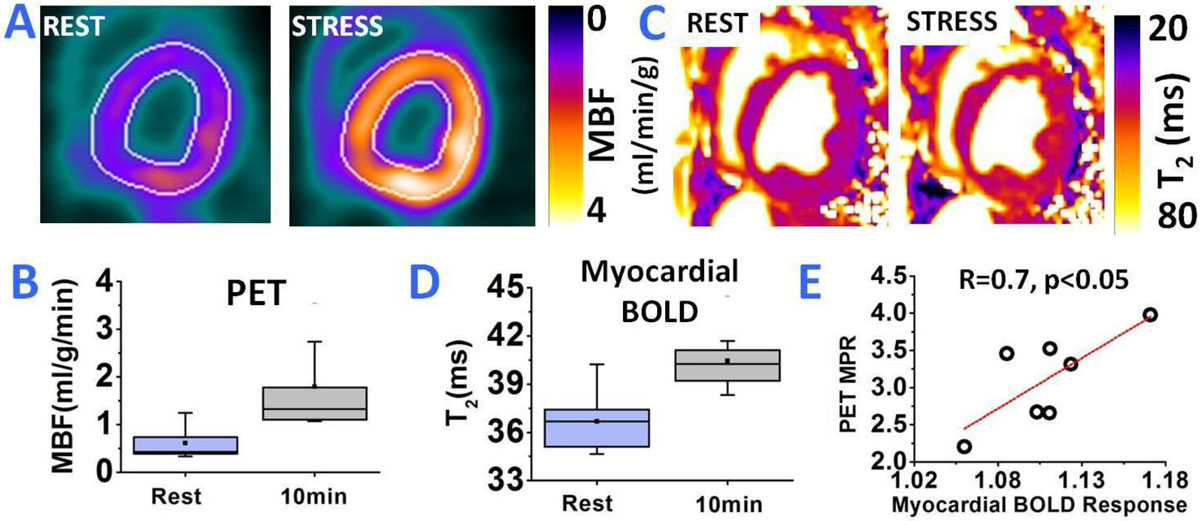
***13N-Ammonia PET Myocardial Blood Flow and BOLD Response at 10 mins post regadenoson administration (p.r.a.)***. Representative rest and stress (10 mins p.r.a) short-axis PET Myocardial Blood Flow (MBF) and myocardial BOLD T_2_ pages are shown in panels A and C. Both PET and BOLD images shown significant increase in MBF and BOLD response, respectively, at 10 mins p.r.a. compared to rest. Box plot of rest and stress MBF and myocardial T_2_ across all animals are shown in panels B and D, respectively. Mean increase in MBF by a factor of 3.0 and a 9% T_2_ elevation were observed on the PET and T_2_ maps acquired at 20 min p.r.a. relative to rest. Results from regression analysis showed good correlation between PET myocardial perfusion reserve (MBF(stress)/MBF(rest)) and Myocardial BOLD Response (R = 0.7, p < 0.05; panel E).

A box-plot of observed MBV at 2- and 10-mins p.r.a (and at rest, for reference), along with representative T_2_ maps are shown in Fig. 1. Note the extensive artifacts present in the T_2_ map at 2 min, which are absent in the T_2_ maps acquired at rest and 10-mins p.r.a. MBV was significantly larger at 2-mins p.r.a (1.6 ± 0.9) compared to 10-mins p.r.a (1.0 ± 0.3) and rest (1.0); p < 0.05 for both. Representative MBF at rest and 10-mins p.r.a are shown in Fig. 2A. MBF at 10-min p.r.a (1.8 ± 0.9 ml/g/min) was significantly higher than at rest (0.6 ± 0.3 ml/g/min), p < 0.05 (Fig. 2B). Mean MPR at 10-min p.r.a was 3.0. Corresponding BOLD images (T_2_ maps) are shown in Fig. 2C. Myocardial T_2_ at 10-min p.r.a (40.4 ± 1.7 ms) was significantly higher than at rest (37.1 ± 2.0 ms), p < 0.05 (Fig. 2D). MBR was strongly correlated with MPR (R = 0.7, p < 0.05, Fig. 2E)

## Conclusions

Myocardial BOLD images acquired at 10-min p.r.a (compared to 2-min p.r.a) can be free of image artifacts. MPR at 10-mins p.r.a can be consistently higher than 2.0 and is strongly correlated with MBR. These data support that delayed acquisition of BOLD CMR post regadenoson administration is a viable means for increasing the reliability of cardiac BOLD. The clinical utility of this approach remains to be evaluated in human subjects.

